# Chorioallantoic membrane assay revealed the role of TIPARP (2,3,7,8-tetrachlorodibenzo-p-dioxin-inducible poly (ADP-ribose) polymerase) in lung adenocarcinoma-induced angiogenesis

**DOI:** 10.1186/s12935-023-02870-5

**Published:** 2023-02-25

**Authors:** Kenji Miura, Michiyo Koyanagi-Aoi, Yoshimasa Maniwa, Takashi Aoi

**Affiliations:** 1 Division of Stem Cell Medicine, Graduate School of Medicine, Kobe University, 7-5-1 Kusunoki-cho, Chuo-Ku, Kobe, Hyogo 650-0017 Japan; 2 Division of Advanced Medical Science, Graduate School of Science, Technology and Innovation, Kobe University, 7-5-1 Kusunoki-cho, Chuo-ku, Kobe, Hyogo 650-0017 Japan; 3 Division of Thoracic Surgery, Graduate School of Medicine, Kobe University, 7-5-1 Kusunoki-cho, Chuo-ku, Kobe, Hyogo 650-0017 Japan; 4 Center for Human Resource Development for Regenerative Medicine, Kobe University Hospital, 7-5-2 Kusunoki-cho, Chuo-ku, Kobe, Hyogo 650-0017 Japan

**Keywords:** Chorioallantoic membrane, Lung cancer, Angiogenesis, Extracellular matrix, 2,3,7,8-tetrachlorodibenzo-p-dioxin-inducible poly (ADP-ribose) polymerase (TIPARP)

## Abstract

**Background:**

The chorioallantoic membrane (CAM) assay is a well-established technique to evaluate tumor invasion and angiogenesis and may overcome the shortcoming of the patient-derived xenograft (PDX) mouse model. Currently, few reports have described lung cancer invasion and angiogenesis in the CAM assay. We therefore used the CAM assay in the evaluation of lung cancer.

**Method:**

Lung cancer cell line-derived organoids or lung cancer cell lines were transplanted into the CAM on embryonic development day (EDD) 10, and an analysis was performed on EDD 15. Microscopic and macroscopic images and movies of the grafts on the CAM were captured and analyzed. The relationships between the graft and chick vessels were evaluated using immunohistochemistry.

**Results:**

We transplanted lung cancer cell lines and cell line-derived organoid into a CAM to investigate angiogenesis and invasion. They engrafted on the CAM at a rate of 50–83%. A549-OKS cells showed enhanced cell invasion and angiogenesis on the CAM in comparison to A549-GFP cells as was reported in vitro. Next, we found that A549-TIPARP cells promoted angiogenesis on the CAM. RNA-seq identified 203 genes that were upregulated more than twofold in comparison to A549-GFP cells. A pathway analysis revealed many upregulated pathways related to degradation and synthesis of the extracellular matrix in A549-TIPARP cells.

**Conclusions:**

The CAM assay can be used to evaluate and research invasion and angiogenesis in lung cancer. The elevated expression of TIPARP in lung cancer may induce angiogenesis by remodeling the extracellular matrix.

**Supplementary Information:**

The online version contains supplementary material available at 10.1186/s12935-023-02870-5.

## Introduction

The development of molecular targeted drugs and immune checkpoint inhibitors (ICIs) has radically altered the treatment of non-small cell lung cancer (NSCLC) [1]. For instance, the epidermal growth factor receptor (EGFR) is one of the driver mutations of NSCLC, and patients with advanced NSCLC have been treated with tyrosine kinase inhibitors targeting EGFR (EGFR-TKI) [2]. The tumor microenvironment has been another focus of research. For example, ICIs targeting the PD-1/PD-L1 in NSCLC patients have resulted in an increase in long-term overall survival (OS) [3, 4]. Moreover, bevacizumab, which targets the VEGF gene, inhibits new vascular development, and is commonly used in the treatment of NSCLC. However, it is not highly effective for all patients. The problems are that the drug response is limited to a subset of patients, and therapeutic targets involved not only cancer cells but also the tumor microenvironment. In order to overcome these problems, the establishment of patient-derived cancer organoids and drug screening have been reported [5]. However, patient-derived lung cancer organoids are difficult to establish because of normal lung epithelium contamination [6]. In addition, because the cancer organoids are composed of epithelial cells without blood vessels and stroma, they are unable to totally predict the effect of treatment on the tumor microenvironment [7]. Patient-derived xenograft (PDX)-based preclinical mouse models recapitulate the pathological and genetic characteristics of individual tumors and represent patients' heterogeneity and clinical features [8]. However, the low success rate of transplantation remains problematic. In lung cancer PDX, the reported engraftment rates are 33%, 40% and 49% [9–11], and engraftment of squamous cell carcinoma was reported to be more likely to be successful than engraftment of adenocarcinoma [11]. Thus, the utility of the PDX mouse model is heavily dependent on the tumor characteristics. In addition, the human stroma of the PDX can be replaced by murine cells after several passages. Another limitation is that it takes 4–8 months to establish a PDX model for drug screening [6]. Therefore, it is often beyond the time when patients can begin treatment.

In previous reports, co-culturing iPS cells-derived hepatocyte or mouse parenchymal cells of various organs with human umbilical vein endothelial cells (HUVECs) and mesenchymal stem cells (MSCs) resulted in the formation of self-organized spheres [12, 13]. Furthermore, by co-culturing with HUVECs and MSCs, we generated a human lung cancer cell line: “A549-OKS-colony”-derived organoids that mimicked patient-derived lung cancer tissues [14]. Although our organoids were cell line derived, the limitation of PDX models could be overcome. A549-OKS was generated by retroviral transduction of OCT3/4, KLF4, and SOX2 into the human lung cancer cell line (A549) (hereafter A549-OKS). After 7–10 days of transduction, A549-OKS cells formed a colony accompanied by spindle-like cells [14]. In this study, we regard A549-OKS-colony as A549-OKS cells. In comparison to A549 cells, A549-OKS-colony cells showed enhanced cisplatin-resistance, invasion, sphere-formation ability, and tumorigenesis. To reveal the difference between A549-OKS-colony and A549 cells, we carried out gene expression profiles using the Human Gene Expression Microarray (Agilent Technologies). The GO analysis showed that the upregulated genes in A549-OKS cells were associated with “blood vessel development” and “vascular development” in comparison to A549 cells. Notably, the expression of VEGF, which is well-known to be an essential factor in angiogenesis, and its associated genes, were not altered [14].

The chorioallantoic membrane (CAM) assay is reported to be a well-established model for evaluating tumor invasion and angiogenesis [15]. The CAM is a respiratory organ for chick embryos that can be identified from embryonic development day (EDD) 3. The CAM is reported to be immunodeficient with engraftment rates of 100% for nasopharyngeal carcinoma and renal cell carcinoma and 79% for glioblastoma [16]. However, few studies have reported the use of CAM assays in the investigation of lung cancer [17]. Moreover, the CAM assay is considered to be a low-cost experimental system. A fertilized egg costs one hundred times less than an immunodeficient mouse. Thus, we examined the CAM culture and our organoids as a model for researching the development and angiogenesis of lung cancer.

In this report, we established a CAM culture assay as a model for the quantitative evaluation of lung cancer angiogenesis. Using the assay, we found that the overexpression of TIPARP in lung cancer cells enhanced angiogenesis. Moreover, a transcriptome analysis suggested that TIPARP might promote angiogenesis via remodeling of the extracellular matrix.

## Materials and methods

### CAM assay

Fertilized White Julia Light hen eggs were purchased from Japan Layer Inc., (Gifu, Japan). The eggs were incubated in LJEI002 (Life Basis) at 37 °C with 60% humidity. On embryonic development day (EDD) 8, a hole of 3 mm in diameter was made in the eggshell to create an air space between the shell and the CAM. On EDD 10, a hole was extended to approximately 2 × 1 cm^2^ to expose the CAM and wrapped with PARAFILM (Bemis Company Inc. Neenah, Wisconsin, USA) to prevent drying out. Cells were transplanted onto the CAM by suspending them in 100 µL of medium (and dropping them) on EDD 10. The eggs were incubated until EDD 15. Images were captured using an iPhone SE2 (Apple Inc., California, USA). Blood flow was captured using a TOKU Capillaro (Toku Corporation Inc, Tokyo, Japan). Specimens were captured by an Olympus stereomicroscope SZ61 (Olympus Inc., Tokyo, Japan) attached to a WRAYCAM-NOA630 (WRAYMER Inc., Osaka, Japan).

### Cell culture

The human lung adenocarcinoma cell line A549 (RCB0098) and Platinum-A (Plat-A) Retroviral packaging cells were purchased from the RIKEN BioResource Centre and Cell Biolabs, respectively. Both cell lines were incubated in Dulbecco’s modified Eagle’s medium (DMEM, Nacalai Tesque, Kyoto, Japan) with 10% fetal bovine serum (FBS) (Life Technologies, Paisley, U.K), penicillin (50 Units/mL) and streptomycin (50 µg/mL) (Gibco, Carlsbad, CA, USA) at 37 °C under 5% CO_2_. For cultivation of Plat-A cells, 10 µg/mL of blasticidin (Funakoshi, Tokyo, Japan) and 1 µg/mL of puromycin (Nacalai Tesque, Tokyo, Japan) were added. Human mesenchymal stem cells (MSCs) and human umbilical vein endothelial cells (HUVECs) were obtained from Lonza (Lonza, Basel, Switzerland). MSCs were maintained in MSC growth medium (Lonza, Basel, Switzerland) at 37 °C under 5% CO_2_, and used up to passage five. HUVECs were cultured in endothelial growth medium (Lonza, Basel, Switzerland) at 37 °C under 5% CO_2_.

### Co-culture method

To form spheres in vitro, we mixed the cells at the same ratio as in our previous report [14], A549-GFP or A549-OKS: HUVECs: MSCs = 10:1:4. A549-GFP or A549-OKS cells (4.0 × 10^5^), HUVECs (4.0 × 10^4^), and MSCs (1.6 × 0^5^) were mixed and resuspended in sphere-forming medium, which contained 10 ng/mL bFGF (WAKO, Osaka, Japan), 10 μg/mL human insulin (Cell Science & Technology Institute, Sendai, Japan), 100 μg/mL human transferrin (Roche, Basel, Switzerland) and 100 μg/mL BSA (Nacalai Tesque, Kyoto, Japan), and seeded on a low-attachment 24-well flat plate or 96-well M bottom plate (Sumitomo Bakelite, Tokyo, Japan) [14]. After one day of culture, self-organized spheres were formed.

### Drug treatment in the CAM assay

Filter paper was soaked with the phosphate-buffered saline (PBS) containing the drug and placed near the graft on EDD 12. The filter paper was 5.5–6.5 mm in diameter.

### Digital measurement in the CAM assay

The analysis of vessel area and mass area was performed with Image J. All images were captured from the bottom side using an Olympus stereomicroscope SZ61 at 1.5 ×magnification. Images were converted to 8-bit and the color balance was adjusted to clearly show the microvessels. The large vessels that originally existed were excluded from the observation target, but vessels branching from them were measured. The mass area was defined as a thick white area around the graft.

### Construction of pMXs-TIPARP

To generate pMXs-TIPARP, the TIPARP coding region was amplified by PCR using A549-OKS cDNA as a template and cloned into the BamHI/ECoRI restriction sites of pMXs vector using an In-Fusion® cloning kit (Clonetech Laboratories Inc., Mountain View, CA) according to the manufacturer’s protocol.

### Retroviral transduction

We modified previously described methods for retroviral transduction and used pMXs-GFP, pMXs-OKS and pMXs-TIPARP vectors [14, 18]. PLAT-A packaging cells were plated at 1.2 ×10^6^ cells per 6-cm dish and were incubated overnight. The following day, the cells were transfected with pMX vectors using the Fugene HD transfection reagent (Promega Inc, Madison, WI, USA). In addition, pMXs-GFP was used as the transfection control. After 24 h of transfection, the virus-containing supernatant was replaced with fresh medium. The virus-containing supernatants were filtered through a 0.45 μm pore filter and supplemented with 4 μg/mL polybrene (Nacalai Tesque, Kyoto, Japan), transferred to the A549 cancer cell line and incubated overnight.

### RNA isolation

Total RNA was isolated using TRIzol reagent (Life Technologies, Paisley, UK). To remove residual genomic DNA, DNase treatment was performed using a TURBO DNA-free kit or ezDNase enzyme (Thermo Fisher Scientific, Waltham, MA, USA).

### RNA sequencing

RNA was sent to Macrogen (Seoul, South Korea) for library preparation and paired-end RNA sequencing was performed on the Illumina Novaseq 6000 platform. Raw sequence files (fastq) were aligned to the human transcriptome (hg38) reference sequences using the Strand NGS software program (Strand Life Science, Karnataka, India) with default parameters. For the analysis, only the genes whose TPM values were > 2 in at least one sample of six samples were used to filter out noise from the expression data. The total number of genes used in the analysis was 13181. A pathway analysis was performed using the Strand NGS software program. RNA-seq data were deposited into the Gene Expression Omnibus (GEO) database with Accession No. GSE199414.

### Semi-quantitative reverse transcription-polymerase chain reaction

RNA (250 ng) was reverse transcribed into cDNA using a Prime Script II 1st strand cDNA Synthesis Kit (Takara Bio, Shiga, Japan). Semi-quantitative reverse transcription-polymerase chain reaction (RT-PCR) was conducted using Ex Taq (Takara Bio, Shiga, Japan) on a PCR Thermal Cycler Dice Touch (Takara Bio, Shiga, Japan). The PCR products were visualized by 2% agarose gel electrophoresis stained with ethidium bromide. The primer sequences were as follows: GAPDH forward primer, 5′-agccacatcgctcagacac-3′; GAPDH reverse primer, 5′-gcccaatacgaccaaatcc-3′; TIPARP forward primer, 5′-gtccctgtttctgcagagga-3′; TIPARP reverse primer, 5′-atcctgtcacggccaaacat-3′.

### Hematoxylin and Eosin staining and immunohistochemistry

The CAM with the transplanted graft was embedded in paraffin blocks and cut into 6-μm-thick sections. The sections were deparaffinized and stained with hematoxylin and eosin (HE). For immunohistochemistry, the sections were stained with anti-Desmin mouse monoclonal antibody (clone: ab8470, dilution 1:50, Abcam, Cambridge, MA, USA), anti-CD31 rabbit polyclonal antibody (clone: ab28364, dilution 1:80, Abcam, Cambridge, MA, USA), and anti-ASCT2 rabbit polyclonal antibody (clone: HPA035240, dilution 1:50, Sigma-Aldrich, St Louis, MO, USA).

### Statistical analysis

All statistical analyses were performed using the Prism software program (GraphPad Software, USA). Differences in mean values between two groups were determined using paired or unpaired *t*-tests. *P* values of < 0.05 were considered to indicate statistical significance (* *P* < 0.05, ** *P* < 0.01, and *** *P* < 0.001).

## Results

### CAM culture as a model for researching lung cancer angiogenesis

To examine whether the chorioallantoic membrane (CAM) culture can be used as a research model of lung cancer, we used a 3D co-culture system of cell line-derived organoids that recapitulated human lung cancer tissues (Additional file 1: Fig. S1a) [14]. The organoid consisted of A549-OKS cells, human umbilical vein endothelial cells (HUVECs) and mesenchymal stem cells (MSCs).

**Fig. 1:**
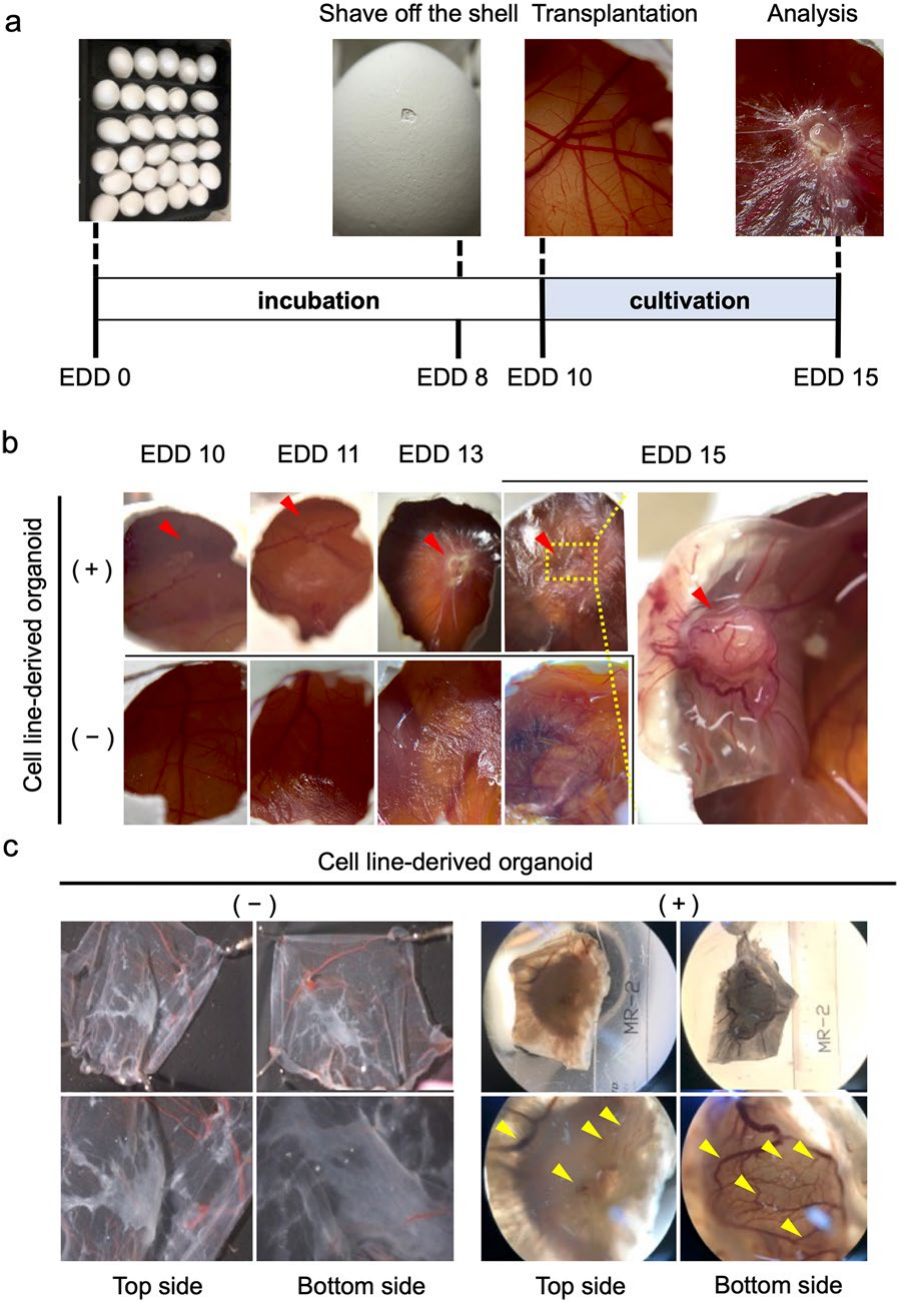
The chorioallantoic membrane (CAM) assay as a model for researching lung cancer engraftment and angiogenesis. **a**. A schematic diagram of the CAM assay. **b**. Macroscopic images are captured on embryonic development day (EDD) 10, 11, 13 and 15. A cell line-derived organoid engrafts the CAM and induces angiogenesis (upper panels). The red arrowheads indicate transplanted organoids. The right image shows an enlarged bottom view of the area enveloped by the yellow dotted lines. The sphere formation medium does not recruit the vessels (lower panels). **c**. Microscopic images show graft-induced angiogenesis in the CAM assay on EDD 15. A cell line-derived organoid recruited microvessels from thick vessels (right panels). Upper and lower panels were captured at 0.67 × and 1.5 × magnification. Yellow arrowheads indicate new vessels on the mass surface

Since the CAM, which is a respiratory organ for chick embryos, rapidly developed from embryonic development day (EDD) 10 to 15 (Additional file 1: Fig. S1b), we used a strategy based on the previously reported CAM assay, as illustrated in Fig. 1a [15, 16, 19]. We transplanted cell line-derived organoids onto the CAM on EDD 10 (Additional file 1: Fig. S1c) and examined whether or not they were grafted five days after implantation. Even though we had no prior experience in performing the CAM assay, we were able to successfully reproduce the previously reported CAM preparation and transplantation methods. The results showed that engraftment on the CAM was observed in 10 of 12 trials (83%) (Fig. 1b and data not shown).

Tumor cells stimulate the stroma and blood vessels to perform angiogenesis in order to supply nutrients and oxygen to themselves and grow and CAM culture is a well-established platform for evaluating tumor angiogenesis [15, 16, 19], which is defined as new vascular networks generated from preexisting vessels [20]. A closer look around the site where we transplanted the cell line-derived organoids revealed that new tortuous and dilated vessels were formed at the graft surface on the CAM (Fig. 1c right panels). We confirmed that the addition of sphere formation medium (Fig. 1b lower panels, 1c left panels) alone did not cause such abnormal vascularization.

Next, we investigated the interaction between chick vessels and transplanted cell line-derived organoids in our system by immunohistochemistry (IHC). We used Desmin as a smooth muscle marker to detect vessels [21] and Amino acid transporter type 2 (ASCT2) to distinguish human organoids from chicken tissues. ASCT2 is expressed only in mammals, not in birds [22, 23]. IHC to detect cell line-derived organoids graft onto the CAM revealed that the ASCT2-positive organoids invaded the CAM and were surrounded by Desmin-positive vessels (Additional file 2: Fig. S2a). Furthermore, we analyzed whether these vessels were derived from human or chicken using CD31 antibodies that recognize human vascular endothelium. Pre-transplant organoids in vitro contained CD31-positive cells (Additional file 2: Fig. S2b left panel) [21], but not Desmin-positive cells (Additional file 2: Fig. S2b right panel). However, organoids on the CAM after transplantation did not contain CD31-positive cells (Additional file 2: Fig. S2c). These results suggested that our transplanted organoids were supplied by chick vessels alone.

### Invasion and angiogenic ability of A549-OKS cells in the CAM assay

**Fig. 2:**
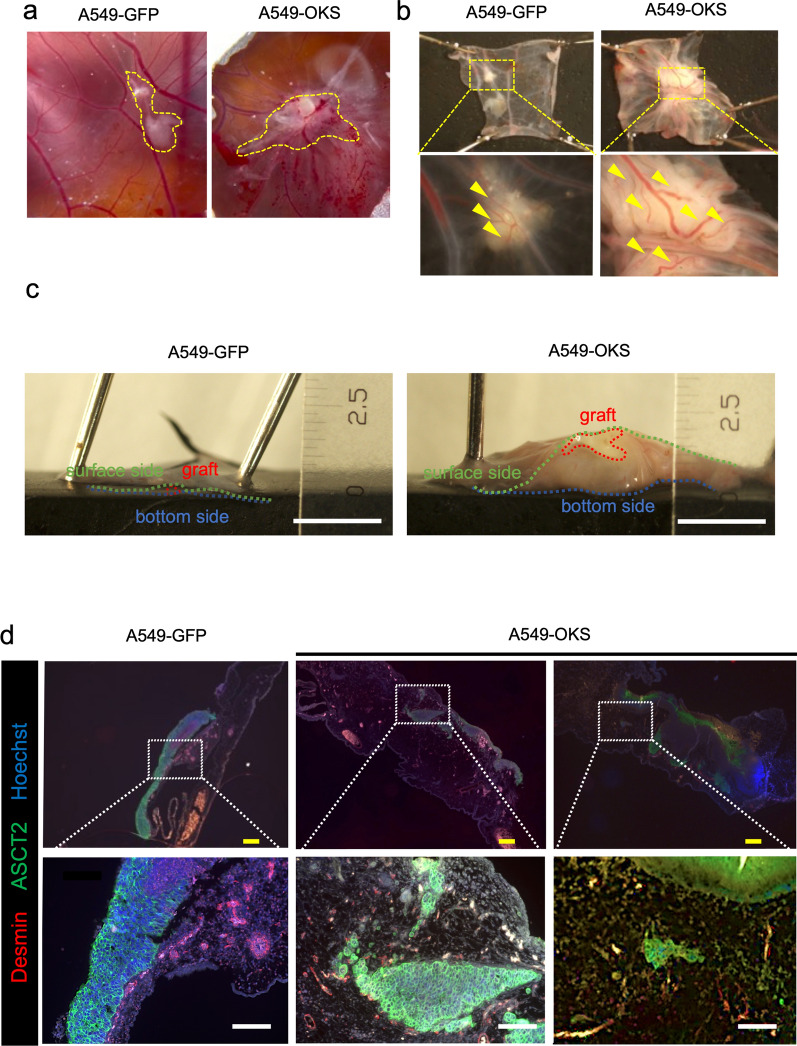
Invasion and angiogenesis of A549-OKS cells on the CAM. **a**. Macroscopic images of CAM engrafted with A549-GFP (left panel) and A549-OKS cells (right panel) on embryonic development day (EDD) 15. The part surrounded by the yellow dotted line indicates the grafts. **b**. Microscopic images of CAM engrafted with A549-GFP and A549-OKS cells on EDD 15. Linear blood vessels are distributed on the surface of the A549-GFP graft. A549-OKS cells make thick vessels twist and induce microvessels. Upper and lower panels are captured in 0.67 and 1.5 × magnification. Yellow arrowheads indicate capillary vessels on the transplanted graft. **c**. A cross-section view of Fig. 2 b. The right panel shows an enlarged image of A549-OKS cells on the CAM. A549-OKS cells invade the CAM, and the surrounding stroma is thickened. The green, blue, and red dotted lines indicate the surface, bottom, and transplanted graft, respectively. Scale bars: 2.5 mm. **d**. Immunohistochemistry (IHC) to detect Desmin and ASCT2 of CAM with A549-GFP or A549-OKS cells on EDD 15. Desmin-positive cells (shown in red) and ASCT2-positive cells (shown in green) indicate the chick vessels and the transplanted cells, respectively. The grafts are on the membranes in A549-GFP (left panels) and induce a few vessels. The central panel shows that A549-OKS cells with microvessels invade the membrane. The right panel shows cells located away from the mass. Hoechst was used to stain nuclei. Scale bars: 100 μm

We have previously shown that A549-OKS cells have the ability to promote invasion in comparison to A549-GFP cells in a double-layer invasion assay [14]. Moreover, it was also shown that only A549-OKS cells can form spheres containing HUVEC cells in mixed culture with MSC and HUVEC cells [14]. We investigated whether these in vitro properties of A549-OKS cells could be recapitulated in vivo as the ability of “invasion” and “angiogenesis” by transplantation of only A549-OKS or A549-GFP cells into the CAM and observed the direct interaction between human cancer cells and chicken stroma. The survival rate of the eggs in medium only, A549-GFP, and A549-OKS was 50% (8 of 16 trials), 70.6% (12 of 17 trials) and 57.1% (12 of 21 trials), respectively. Moreover, grafts of A549-GFP and A549-OKS cells were observed in 50% (6 of 12 trials) and 66.6% (7 of 12 trials) on EDD 15, respectively (Fig. 2a).

Since A549-OKS cells showed the increased expression of genes related to the GO terms of “blood vessel development (GO: 0001568)” and “vasculature development (GO: 0001944)”, in comparison to A549-GFP cells in our previous report [14], we investigated the A549-OKS cell-induced angiogenetic activity using the CAM assay. The results revealed that A549-OKS cells twisted pre-existing vessels and induced tortuous vessels. In contrast, A549-GFP cells recruited small linear vessels (Fig. 2b lower panels). Hence, the blood flow was magnified and observed using a previously reported technique [24]. The TOKU Capillaro allowed us to visualize the movement of red blood cells in vessels and arterial and venous blood flows from the mass, which could not be confirmed with conventional tissue specimens or under microscope. In the non-transplantation group, straight-like vessels branching from the thin vessels were observed (Additional file 8: Video S1). In A549-GFP cells, the blood vessels were tortuous and fed into the mass (Additional file 9: Video S2). A549-OKS cells formed a white mass that was covered with tortuous blood vessels. These vessels showed irregular blood flow and formed a capillary network. Furthermore, we found that the red blood cells, which drained from the graft, flowed to the graft through this capillary network again (Additional file 10: Video S3). It was noteworthy that we could confirm the graft-induced angiogenesis and complex blood flow at magnification.

The cross-sectional image of the area around the graft revealed the graft invasion and peri-tumoral edema of the chicken stroma surrounding the graft only when A549-OKS cells were transplanted (Fig. 2c). To confirm the interaction between A549-GFP or A549-OKS cells and chick vessels, we performed immunohistochemical staining of the area around the graft. When A549-GFP cells were transplanted, ASCT2-positive grafts were found above the CAM and did not invade the CAM (Fig. 2d left panels). Furthermore, Desmin-positive vessels were observed mainly near the grafts. In contrast, A549-OKS (ASCT2-positive) cells invaded the CAM and were covered by Desmin-positive blood vessels. Some cells were located away from the invasive area (Fig. 2d middle and right panels). These results may indicate that A549-OKS cells recruited vessels for growth and invasion. The enhanced features of A549-OKS cells were recapitulated in the CAM assay.

### Quantification of angiogenic activities on the CAM

Next, we aimed to quantify the in vivo angiogenic activity on the CAM engrafted with lung cancer cells with Bevacizumab treatment [25, 26], which is known to inhibit VEGF-induced vascularization. First, we confirmed that no antiangiogenic effect and no abnormal vessels were observed in control embryos implanted with filter paper soaked in phosphate-buffered saline (PBS) alone (Additional file 3 Fig. S3a). We placed filter paper containing Bevacizumab (12.5 μg) on the CAM at EDD 12 [27] and found the inhibition of chick microvessel formation around the paper after two days (Additional file 3: Fig. S3b).

**Fig. 3:**
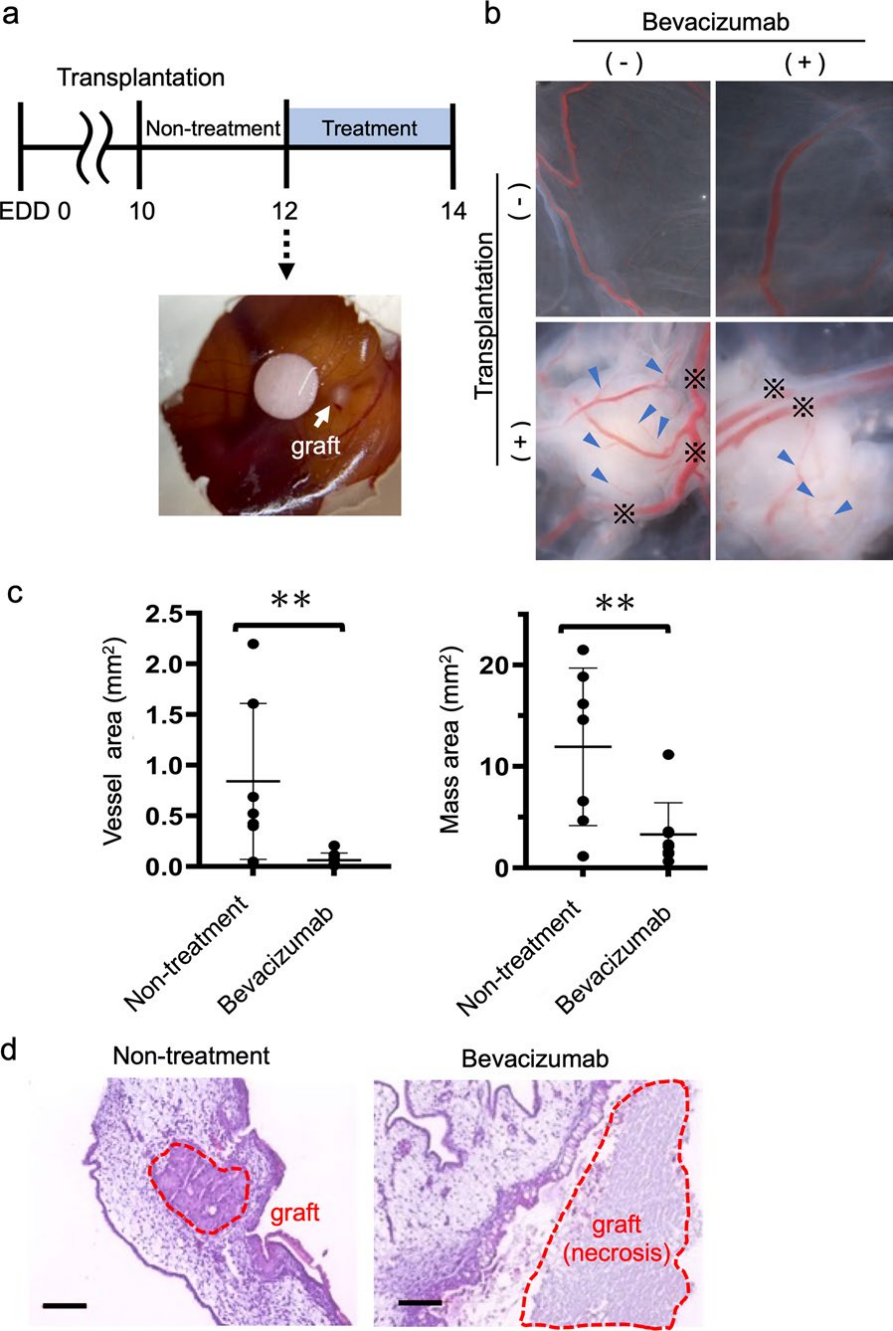
Quantification of angiogenic activities in the CAM assay. **a**. A schematic diagram of treatment with Bevacizumab in the CAM assay. The filter paper soaked with phosphate-buffered saline (PBS) containing Bevacizumab was placed near the graft on embryonic development day (EDD) 12 and the transplant site was observed two days later. **b**. Macroscopic images from the bottom side. Bevacizumab inhibited tumor angiogenesis. Lower and upper panels show CAMs with and without transplantation. Pictures were captured at 1.5 × magnification. Blue arrowheads indicate the blood vessels that we measured. Blood vessels marked with “※” were preexisting vessels and were excluded from the measurements. **c.** Dot plots of the vessel area (mm^2^) and mass area (mm^2^) measured by Image J. Bevacizumab reduced the vessel area. The left graph shows the vessel area. The right graph shows the mass area. Non-treatment; n = 7, Bevacizumab; n = 9. Mean values ± SE, unpaired *t* test, **P < 0.01. **d**. Hematoxylin and eosin (HE) staining is shown on EDD 14. The part surrounded by the red dotted line is the graft. Scale bars: 100 μm

Based on these results, we set the timing of transplantation and Bevacizumab treatment as shown in Fig. 3a. We implanted the organoids onto the CAM on EDD 10, put the filter paper soaked with or without Bevacizumab near the graft on EDD 12 and observed the vessel formation on EDD 14. In the non-transplantation group, the effect of Bevacizumab on microvessels was unclear (Fig. 3b upper panels). In the non-treatment group, chick vasculature penetrated the tumor graft and new vessels (indicated with blue arrowheads) were visible at the surface of the tumor grafts (Fig. 3b left lower panel). In contrast, the number of newly-formed macroscopic blood vessels was reduced in Bevacizumab-treated group (Fig. 3b right lower panel).

Next, we quantitated the in vivo angiogenic activity by measuring the area of neo-vessels and mass area using Image J (Additional file 3: Fig. S3c) [28–30]. All images for measurements are shown Additional file 3: Fig. S3d. In the Bevacizumab-treated group, there was a significant decrease in vessel area (p = 0.0087) and mass area (p = 0.0085) in comparison to the non-treatment group (Fig. 3c).

In the blood flow analysis, the TOKU Capillaro exhibited arterial and venous blood vessels that flowed around the graft and formed capillary webs in the non-treatment groups (Additional file 11: Video S4). In contrast, Bevacizumab treatment inhibited the formation of microvessels, as shown in the macroscopic image (Additional file 12: Video S5). As we expected, Bevacizumab inhibited tumor-induced microvessels on the CAM.

Histologically, hematoxylin and eosin (HE) staining of CAM sections showed necrotic grafts in the Bevacizumab-treated group (Fig. 3d). This indicated that the grafts on the CAM were supported by the chick vessels and could not survive when their formation was inhibited. In conclusion, we established a simple method to quantify the changes in vascular distribution on the CAM by microscopic imaging.

### A549-TIPARP cells induced angiogenesis in the CAM

In a previous study, we found the expression of genes involved in “blood vessel development” and “vasculature development” was increased by the introduction of OCT3/4, KLF4 and SOX2 into A549 cells by a GO analysis [14]. Among these genes, we focused on 2,3,7,8-tetrachlorodibenzo-p-dioxin-inducible poly (ADP-ribose) polymerase (TIPARP) [31], which mediates mono-ADP ribosylation and was reportedly upregulated in A549 under P.M 2.5 exposure [32]. Since there are no reports of its association with lung cancer-angiogenesis, we investigated whether TIPARP is involved in the enhancement of angiogenesis by the introduction of three lung cancer-related factors using CAM assay.

**Fig. 4:**
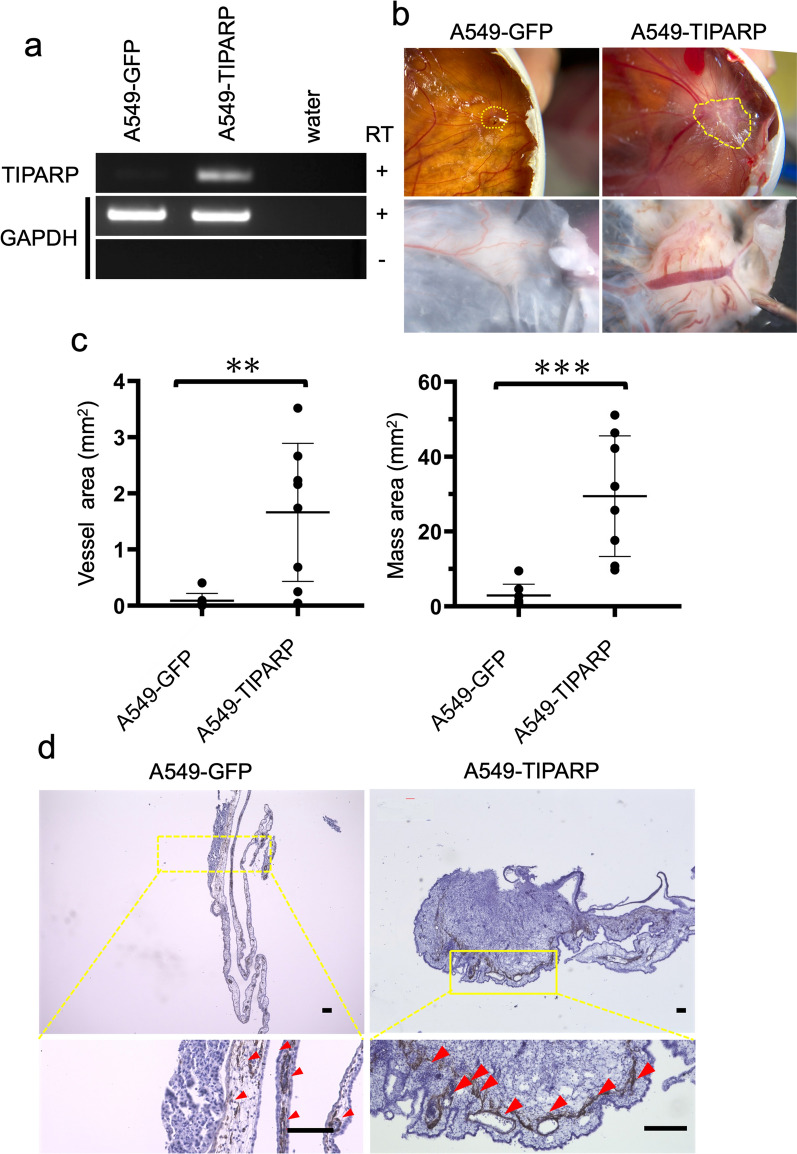
Transplantation of A549-TIPARP cells induced angiogenesis in the CAM assay. **a**. Confirmation of the overexpression of TIPARP in A549 cells by semi-quantitative RT-PCR. GAPDH is used as a loading control. Water is used as a negative control. RT, reverse transcriptase. **b**. Macroscopic (upper panels) and microscopic (lower panels) images of CAM engrafted with A549-GFP cells and A549-TIPARP cells on EDD 15. A549-TIPARP cells induce angiogenesis on the CAM. Yellow dotted lines enveloped the mass. **c**. The vessel area (mm^2^) and mass area (mm^2^) were measured by Image J and are shown as dot plots in the left panel and right panel, respectively. The results were obtained from eight independent experiments. Mean values ± SE, unpaired t test **P < 0.01 and ***P < 0.001. **d**. Immunohistochemistry (IHC) to detect the Desmin expression of CAM engrafted with A549-GFP cells (left panels) and A549-TIPARP cells (right panels) on EDD 15 to stain chick vessels. Higher magnification images of the region outlined by yellow dotted lines in upper panels are shown in the lower panels. Red arrowheads indicate Desmin-positive chick vessels. Scale bars: 100 μm

First, we generated a stable line by overexpressing TIPARP in A549 cells using a retroviral vector and confirmed that the mRNA level of TIPARP was elevated in TIPARP-transduced A549 cells (A549-TIPARP) in comparison to A549-GFP cells, based on semi-quantitative RT-PCR (Fig. 4a). Cell morphologies were not changed by the transduction of TIPARP (Additional file 4: Fig. S4a).

Next, we engrafted A549-TIPARP cells and A549-GFP cells onto the CAM. A549-TIPARP cells were found on the CAM, thickened with microvessels on EDD 15 (Fig. 4b right panels). In comparison to the slight convergence of blood vessels in A549-GFP cells (Fig. 4b left panels), a robust neovascular response converging toward the graft was observed when A549-TIPARP cells were transplanted (Fig. 4b right panels). We independently repeated transplantation to CAM three times and the images of each experiment were also shown in Additional file 4: Fig. S4b. When the vessel area and the mass area were quantified, it was found that both were significantly increased when A549-TIPARP cells were transplanted (p = 0.0029 and 0.0004, respectively). (Fig. 4c). In the blood flow analysis, the graft of A549-TIPARP cells was covered with blood vessels and the arterial and venous vessels were connected (Additional file 13: Video S6), similarly to when A549-OKS cells were transplanted.

The IHC analysis of the CAM showed A549-TIPARP cells induced many Desmin-positive vessels, in comparison to A549-GFP cells (Fig. 4d). It shows that IHC could recapitulate the distribution of blood vessels in the macroscopic images. These results indicate that TIPARP was an important gene for angiogenesis in A549-OKS.

### TIPARP enhances genes related to the remodeling of extracellular matrix

**Fig. 5:**
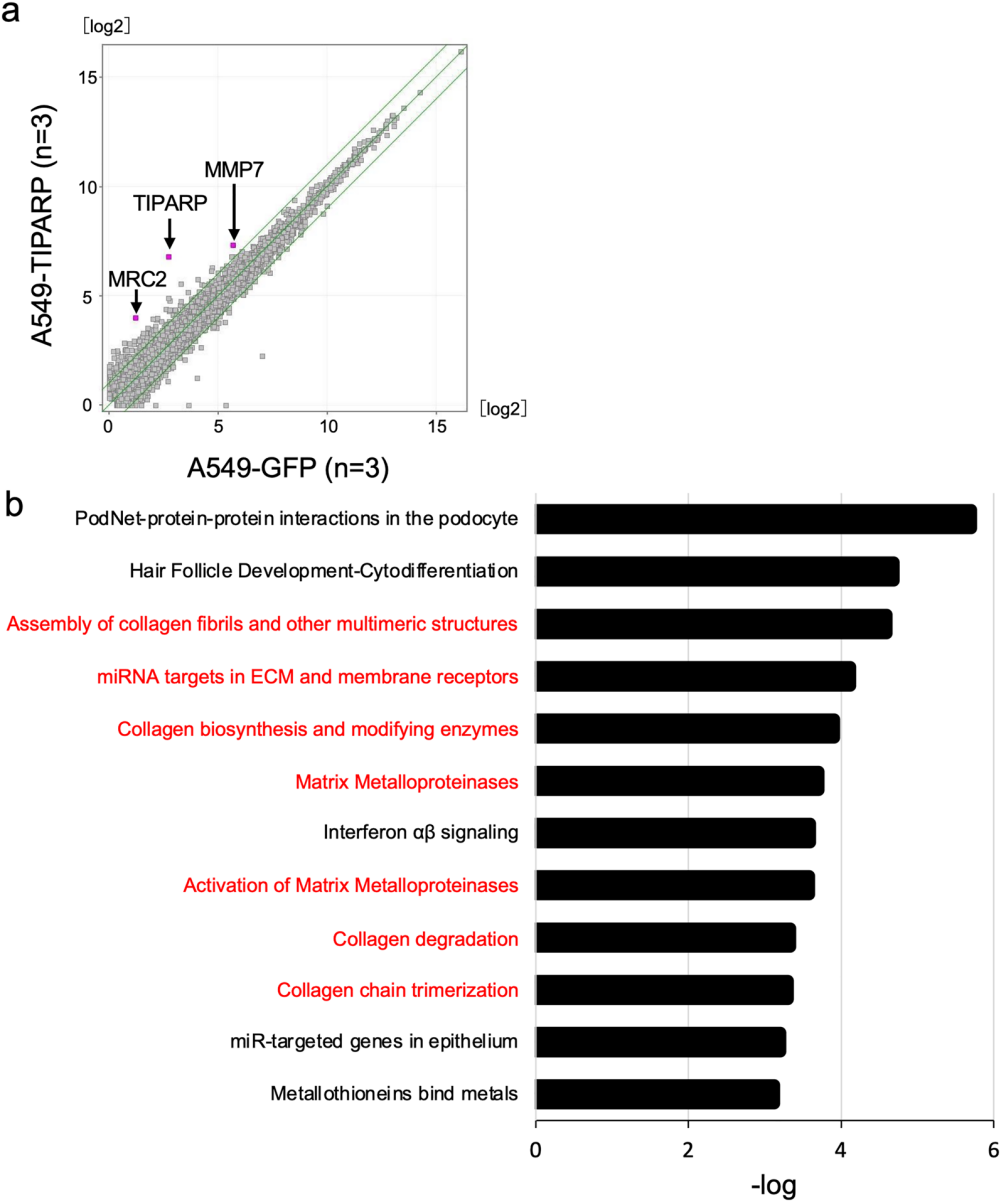
TIPARP enhances genes related to the remodeling of the extracellular matrix. **a**. Scatter plot of the mRNA expression comparing the average values of A549-TIPARP cells (n = 3, y-axis) to the average values of A549-GFP cells (n = 3, x-axis). The RNA expression value is shown on a log 2 scale. Green lines indicate a two-fold differences in the expression level. TIPARP, MRC2 and MMP7 are shown in magenta. **b**. A pathway analysis for upregulated genes (fold-change > 2) in A549-TIPARP cells was performed. The top twelve pathways (*P* < 0.0001) are shown. Pathways related to the extracellular matrix are shown in red

To reveal TIPARP-dependent molecular networks in angiogenesis, we compared the global gene expression profiles of A549-TIPARP cells and control A549-GFP cells. A scatter plot showed the overexpression of TIPARP in A549 cells with 17.2-fold increase, on average, in comparison to control (Fig. 5a). In A549-TIPARP cells, we identified 202 upregulated genes and 57 downregulated genes with a more than twofold change (Additional file 5 and 6: Table S1 and S2, respectively). Among the upregulated genes, mannose receptor C type 2(MRC2), which showed the second highest fold change (= 7.1) after TIPARP, is known to be involved in extracellular matrix (ECM) remodeling and showed an increased expression level with good reproducibility [33]. Moreover, we performed a pathway analysis for 202 upregulated genes and found twelve significant pathways (p < 0.001, Fig. 5b).

Among these twelve pathways, seven (red-colored in Fig. 5b) were related to the extracellular matrix (ECM) and four were related to ECM degradation and remodeling (Fig. 5b): assembly of collagen fibrils and other multimeric structures, collagen biosynthesis and modifying enzymes, collagen degradation, and collagen chain trimerization.

Matrix metalloproteinase 7 (MMP7), which is upregulated in A549-TIPARP cells, is involved in three pathways: matrix metalloprotease, activation of matrix metalloprotease, and collagen degradation. On the other hand, the pathway analysis for 57 downregulated genes showed 18 significant pathways (p < 0.001) (Additional file 7: Table S3), including pro-inflammation cytokine associated pathways.

From these results, TIPARP might promote angiogenesis via the remodeling of the extracellular matrix and inhibition of pro-inflammation cytokine signaling on the CAM.

## Discussion

In the present study, we transplanted cell line-derived organoids to CAM for lung cancer research, and could capture the engraftment, invasion and complexity of the blood vessels surrounding the graft at high magnification, where we could observe individual capillaries and blood cells. While the density of blood vessels on the CAM has been simply assessed in lung cancer [17], this is the first study to report the dynamic visualization and assessment of angiogenesis. Notably, vascular cells contained in our cell line-derived lung cancer organoids neither survived nor connected with the chick's blood vessels on the CAM. Some previous studies have focused on the changes in vascular morphology and calculated the vessel density around grafts [19, 28–31], and argued that human blood vessels survived and were connected to chick vessels when patient-derived renal cancer and glioblastoma tissues containing human vessels were transplanted to a CAM [21, 34]. In the case of cancer metastasis in vivo, the migrating cancer cells do not contain blood vessels, and angiogenesis of the metastatic lesion depends on the induction of blood vessel cells from the tumor microenvironment [35]. Therefore, our system may better recapitulate the angiogenesis of metastatic lesions in vivo in comparison to patient-derived tissue transplantation examinations. Using this system, we confirmed that VEGF inhibitor specifically hindered the angiogenesis of lung cancer, suggesting that this system has potential applicability in the discovery of new selective angiogenesis inhibitors as a quantitative assay to test candidate compounds.

In our previous in vitro study, we demonstrated enhanced characteristics of cell invasion and angiogenesis of A549-OKS cells in comparison to A549 cells [14]. In the present study, we could confirm these characteristics of A549-OKS cells in the CAM assay; A549-OKS cells invaded the CAM with numerous chick vessels on the CAM. In the blood flow analysis, tortuous vessels were connected and covered the grafts. To our knowledge, only one study has revealed the characteristics of angiogenesis and invasion in lung cancer cell lines using CAM assays, and invasion was assessed only by HE staining [17]. One report regarding CAM blood flow without grafting, captured the vessel branches during the development of the CAM [24]. We used ASCT2 antibodies to clearly distinguish between human and chicken cells in IHC, because it is often difficult to distinguish graft invasion and host tissue reaction by HE staining alone. This makes it possible to clearly observe cell invasion into the CAM. Additionally, we calculated vessel density and captured the blood flow at high magnification. It is also important to emphasize that our study captured the blood flow circulating the tumor, connecting blood vessels of various sizes and morphology flowing out of the tumor with those flowing into the tumor. This complexity of blood flow may make the tumor microenvironment suitable for tumor growth. Therefore, the CAM assay is useful for assessing cell invasion and angiogenesis simultaneously within just five days. This report showed the new potential for the application of CAM culture in the study of malignancies.

Our study showed that the overexpression of TIPARP in A549 cells resulted in enhanced angiogenesis on the CAM. TIPARP is found in PM 2.5-exposed A549 cells [32], and the TIPARP locus region on chromosome 3 (3q25) is frequently amplified in squamous cell carcinoma [36]. Regarding the relationship between cancer and the expression of TIPARP, the high expression of TIPARP has been reported to be associated with a poor prognosis and invasion in ovarian cancer [37]. In contrast, in colorectal cancer and breast cancer, the high expression of TIPARP has been reported to be associated with a favorable prognosis [38]. Thus, the functions of TIPARP are not well understood, as it may depend on the cancer characteristics. Furthermore, there are no reports on the relationship between the expression of TIPARP and invasion and angiogenesis in lung cancer. A publicly available database (human protein atlas: http: www.proteiatlas.org) showed that the high expression of TIPARP was associated with a poor prognosis in lung cancer; the 5 year survival rates in high and low expression cases were 38% and 47%, respectively (P = 0.036). Further studies on TIPARP in lung cancer are expected to be conducted in the future.

Our report described, for the first time, the possibility that the high expression of TIPARP induced angiogenesis by degrading and synthesizing the extracellular matrix. MRC2 had the second highest expression in our current data and is reported to be associated with collagen turnover [33]. A pathway analysis revealed multiple significant pathways, including MMP7 and other MMPs. MMPs have been reported to function on various ECM to induce angiogenesis [39, 40]. A previous report identified TIPARP as a target gene of the aryl hydrocarbon receptor (AHR) [41]. Recent research indicates that it is a negative regulator of the hypoxia-inducible factor-1α subunit (HIF-1α) [38], or that it downregulates IFN signaling and evades the immune system [36]. However, the mechanism though which TIPARP induces angiogenesis in cancer remain unclear. The ECM of the CAM contains fibronectin, laminin, and type IV collagen, modulating its composition to promote blood vessel development [42]. Previous studies on ECM have focused on single individual molecules among these ECM components, and no study has evaluated the composite ECMs that are contained in CAM. Therefore, the CAM, which is rich in ECM, may mimic the tumor microenvironment and be applicable as an experimental platform to evaluate the tumor angiogenesis induced by remodeling of the ECM.

The present study was associated with some limitations. (i) Non-specific inflammation occurred and may have masked the phenotype that was truly induced by transplanted tumors. Accordingly, a method using some kind of drug treatment to avoid nonspecific inflammation in CAM after transplantation should be established. (ii) Incomplete chicken-genome information made it difficult to fully understand the host responses. Expansion of information on chicken genes is desired. (iii) It must be verified whether the angiogenic function of TIPARP in chicken eggs can be extrapolated to that in mammals, especially humans. (iv) We only used human lung cancer cell line-derived organoids and did not use patient-derived organoids.

In conclusion, the CAM assay can be used to evaluate and research invasion and angiogenesis in lung cancer. The dynamic blood flow analysis revealed the complexity of the blood flow around the tumor, which could not be analyzed with conventional tissue specimens. Moreover, the elevated expression of TIPARP in lung cancer may induce angiogenesis by remodeling the ECM.

## Supplementary Information

**Additional file 1: Figure S1.** Development of the CAM.** a**. A schematic illustration of co-culture for cell line-derived organoid. A549-OKS cells, MSCs, and HUVECs are resuspended and mixed on a low-attachment plate. After 16 hours of culture, self-organized spheres appear. Scale bars: 500 μm.** b**. Development of CAM from EDD 10 (left panel) to EDD 15 (right panel). CAMs proliferate in five days and surround the embryos. The blue and red dotted lines show the CAM and an embryo, respectively.** c**. Preparation of the CAM assay. To create the space for transplantation, the shells is shaved off on EDD 8 (left panel). After removing the shell, the CAM appears on EDD 10 (right panel). Red arrowhead indicates a transplanted organoid.

**Additional file 2: Figure S2.** The cell line-derived organoid in the CAM. **a**. Immunohistochemistry (IHC) for the detection of Desmin, ASCT2, and Hoechst in CAM with cell line-derived organoids on EDD 15. Transplanted organoids with blood vessels can be seen inside the CAM. Red, green and blue indicate Desmin-, ASCT2-, and Hoechst-positive cells, respectively. Scale bars: 100 μm.** b**. IHC for the detection of CD31 and Desmin in cell line-derived organoids in vitro. The left panels show CD31-positive cells. Brown and yellow arrowheads indicate CD31-positive cells. Desmin-positive cells were not detected (right panels). Scale bars: 100 μm.** c**. IHC for the detection of CD31 of CAM with cell line-derived organoids on EDD15. CD31-positive cells were not found. The right panel shows normal rabbit IgG as a negative control. Scale bars: 100 μm.

**Additional file 3: Figure S3.** Quantification of angiogenesis on the CAM. **a**. Only filter paper was placed as a negative control for two days. The filter paper did not change the vessel shape in the CAM assay on EDD 14. Left and right panels show macroscopic and microscopic images, respectively (magnification: 1.5×). **b**. Bevacizumab inhibited microvessel development on the CAM on EDD14. The left and right panels show non-treatment and treatment with Bevacizumab, respectively. Both images were captured by TOKU Capillaro. **c**. The digital quantification procedure. First, raw image is converted to 8-bit using Image J. Next, the edge of the white areas and the branching vessels was calculated.** d**. All the processed images in Fig. 3c are shown. Non-treatment group, n=6; Bevacizumab group, n=8.

**Additional file 4: Figure S4.** Phenotypes of A549-TIPARP cells *in vitro* and in the CAM. **a**. Phase-contrast and fluorescence microscopy of A549-GFP and A549-TIPARP cells. Black scale bar: 200 μm, gray scale bars: 50 μm. **b**. All images were captured at 1.5× magnification and measured by Image J. n = 8.

**Additional file 5: Table S1.** Two hundred two upregulated genes with a >2-fold change in A549-TIPARP cells.

**Additional file 6: Table S2.** Fifty-seven downregulated genes with a >2-fold change in A549-TIPARP cells.

**Additional file 7: Table S3.** Eighteen downregulated pathways in A549-TIPARP cells.

**Additional file 8: Video S1.** The blood stream of the CAM without transplantation (sphere formation medium). The shape of the blood vessels is straight.

**Additional file 9: Video S2.** The blood stream of the CAM with A549-GFP cells. The white mass is the transplanted cells. Straight vessels run through the graft surface.

**Additional file 10: Video S3. **The blood stream of the CAM with A549-OKS cells. Tortuous vessels showed irregular blood flow and a capillary network was constructed.

**Additional file 11: Video S4.** The blood stream of the CAM with cell line-derived organoids. The organoids induced microvessels.

**Additional file 12: Video S5.** The blood stream of the CAM with cell line-derived organoids treated with Bevacizumab. Bevacizumab inhibited microvessels.

**Additional file 13: Video S6.** The blood stream of the CAM with A549-TIPARP cells. The arterial and venous vessels were connected, similarly to Video S3.

## Data Availability

RNA-seq data have been deposited into the Gene Expression Omnibus (GEO) database with Accession No. GSE199414.
