# Supplementary figures and images for: Chorioallantoic membrane assay revealed the role of TIPARP (2,3,7,8-tetrachlorodibenzo-p-dioxin-inducible poly (ADP-ribose) polymerase) in lung adenocarcinoma-induced angiogenesis
Source: Cancer Cell Int. 2023 Feb 25;23:34. doi: 10.1186/s12935-023-02870-5 (PMC9960622; doi:10.1186/s12935-023-02870-5)

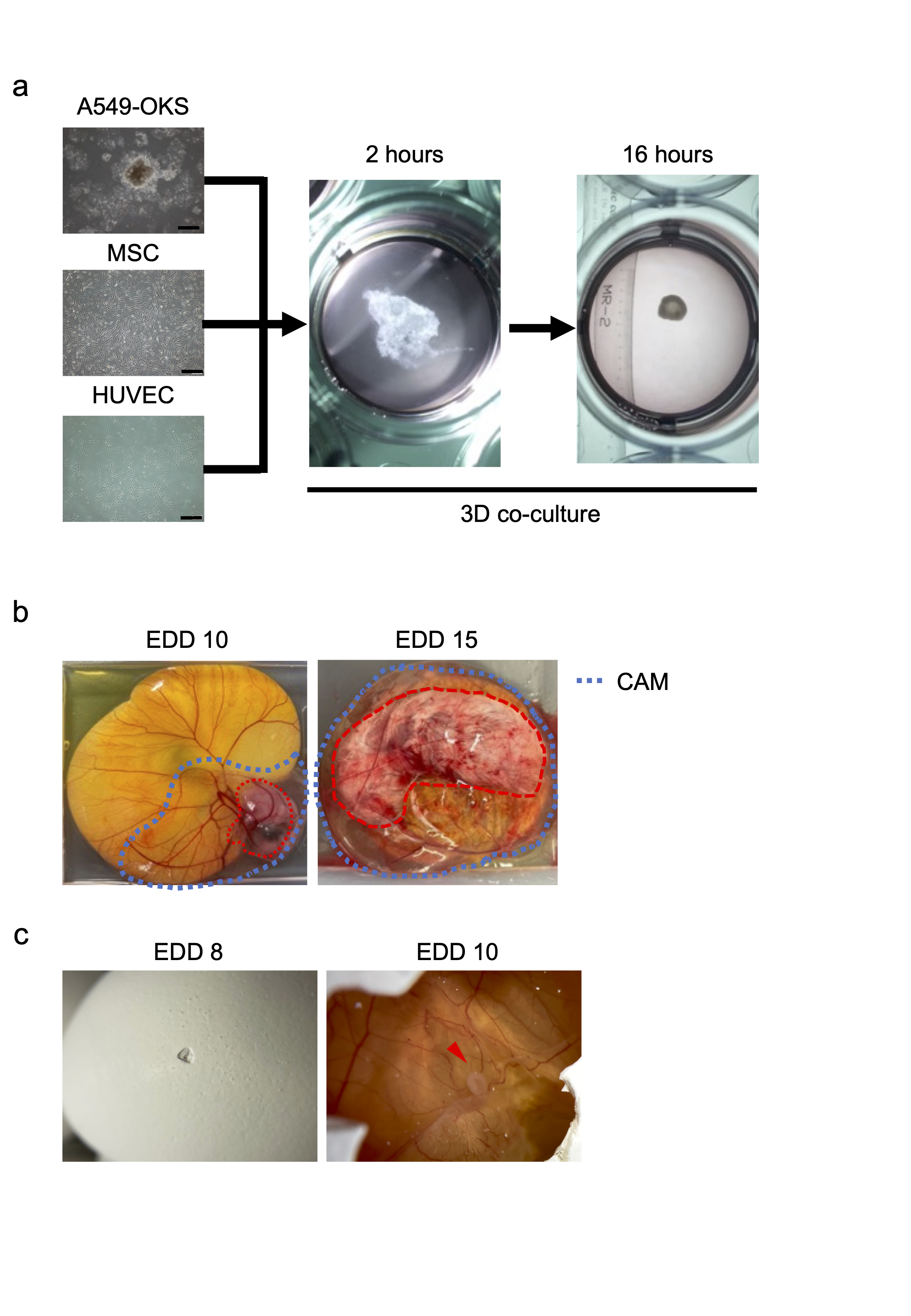

Supplement: Supplementary file 1 — Additional file 1: Figure S1. Development of the CAM. a. A schematic illustration of co-culture for cell line-derived organoid. A549-OKS cells, MSCs, and HUVECs are resuspended and mixed on a low-attachment plate. After 16 hours of culture, self-organized spheres appear. Scale bars: 500 μm. b. Development of CAM from EDD 10 (left panel) to EDD 15 (right panel). CAMs proliferate in five days and surround the embryos. The blue and red dotted lines show the CAM and an embryo, respectively. c. Preparation of the CAM assay. To create the space for transplantation, the shells is shaved off on EDD 8 (left panel). After removing the shell, the CAM appears on EDD 10 (right panel). Red arrowhead indicates a transplanted organoid. [file 12935_2023_2870_MOESM1_ESM.tiff]

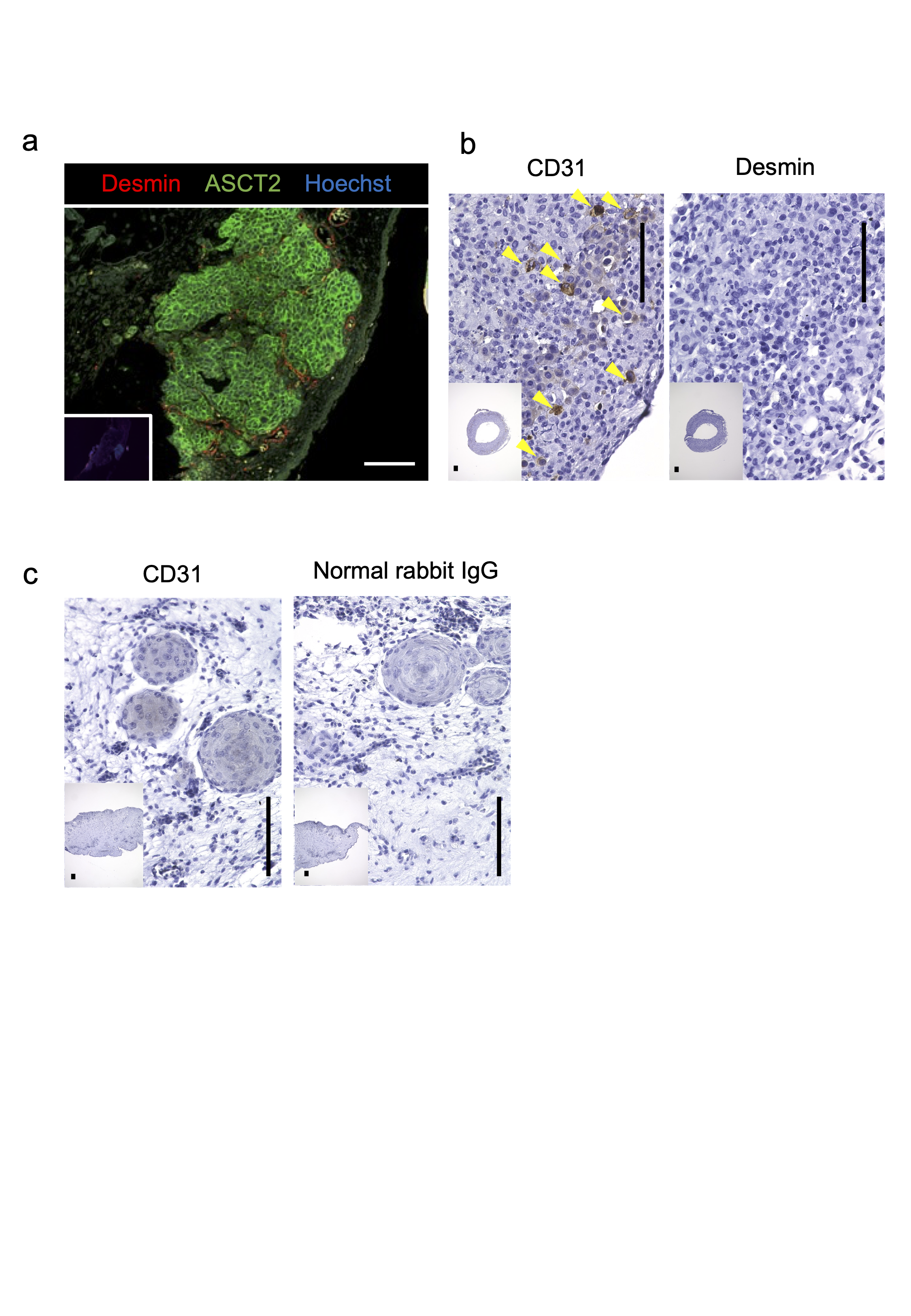

Supplement: Supplementary file 2 — Additional file 2: Figure S2. The cell line-derived organoid in the CAM. a. Immunohistochemistry (IHC) for the detection of Desmin, ASCT2, and Hoechst in CAM with cell line-derived organoids on EDD 15. Transplanted organoids with blood vessels can be seen inside the CAM. Red, green and blue indicate Desmin-, ASCT2-, and Hoechst-positive cells, respectively. Scale bars: 100 μm. b. IHC for the detection of CD31 and Desmin in cell line-derived organoids in vitro. The left panels show CD31-positive cells. Brown and yellow arrowheads indicate CD31-positive cells. Desmin-positive cells were not detected (right panels). Scale bars: 100 μm. c. IHC for the detection of CD31 of CAM with cell line-derived organoids on EDD15. CD31-positive cells were not found. The right panel shows normal rabbit IgG as a negative control. Scale bars: 100 μm. [file 12935_2023_2870_MOESM2_ESM.tiff]

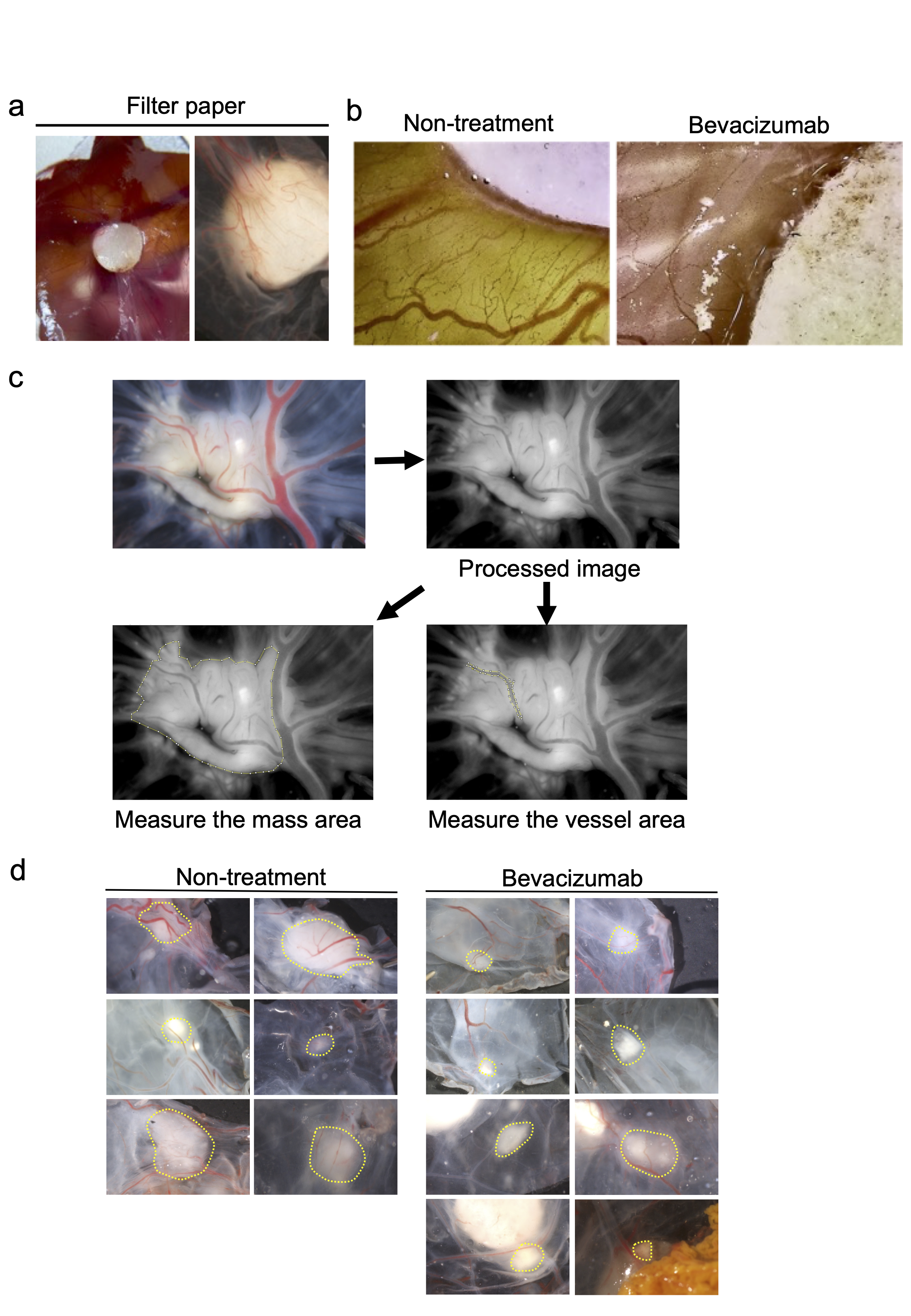

Supplement: Supplementary file 3 — Additional file 3: Figure S3. Quantification of angiogenesis on the CAM. a. Only filter paper was placed as a negative control for two days. The filter paper did not change the vessel shape in the CAM assay on EDD 14. Left and right panels show macroscopic and microscopic images, respectively (magnification: 1.5×). b. Bevacizumab inhibited microvessel development on the CAM on EDD14. The left and right panels show non-treatment and treatment with Bevacizumab, respectively. Both images were captured by TOKU Capillaro. c. The digital quantification procedure. First, raw image is converted to 8-bit using Image J. Next, the edge of the white areas and the branching vessels was calculated. d. All the processed images in Fig. 3c are shown. Non-treatment group, n=6; Bevacizumab group, n=8. [file 12935_2023_2870_MOESM3_ESM.tiff]

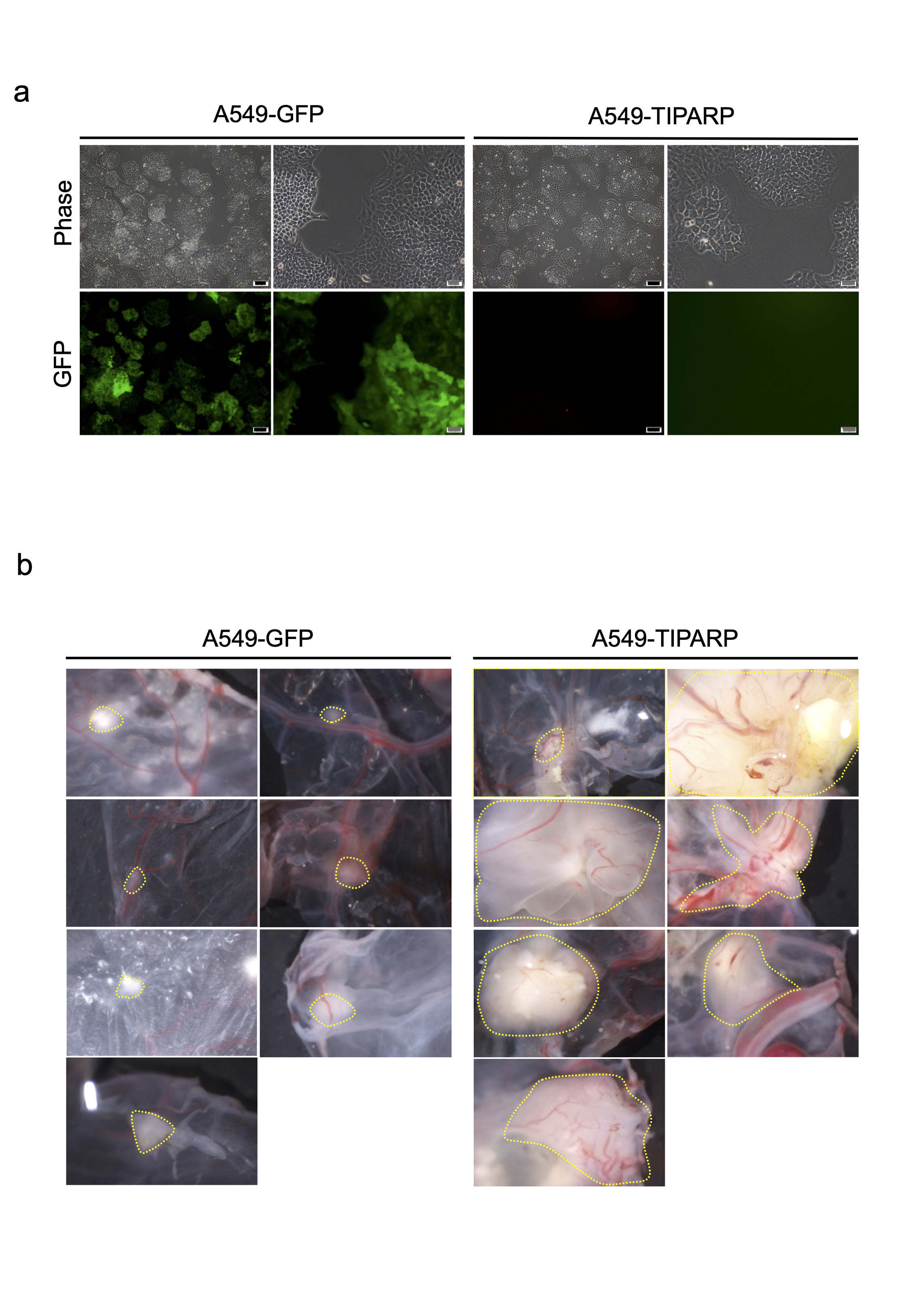

Supplement: Supplementary file 4 — Additional file 4: Figure S4. Phenotypes of A549-TIPARP cells in vitro and in the CAM. a. Phase-contrast and fluorescence microscopy of A549-GFP and A549-TIPARP cells. Black scale bar: 200 μm, gray scale bars: 50 μm. b. All images were captured at 1.5× magnification and measured by Image J. n = 8. [file 12935_2023_2870_MOESM4_ESM.tiff]
